# Higher TYROBP and lower SOX6 as predictive biomarkers for poor prognosis of clear cell renal cell carcinoma: A pilot study

**DOI:** 10.1097/MD.0000000000030658

**Published:** 2022-12-23

**Authors:** Xian-Qiang Lv, Kai-bo Zhang, Xu Guo, Long Pei, Feng Li

**Affiliations:** a Department of Urology, The Fourth Hospital of Hebei Medical University, Shijiazhuang, Hebei Province, China; b Department of plastic surgery, The Fourth Hospital of Hebei Medical University, Shijiazhuang, Hebei Province, China; c Lab of Gambridge Analytica, Heping Road, Shijiazhuang, Hebei Province, China.

**Keywords:** Clear cell renal carcinoma, neural network, survival prognosis, target, TYROBP

## Abstract

Clear cell renal carcinoma (ccRCC) is the most common subtype of renal cancer, accounting for approximately 75% of all histological types of renal cancer, and is the leading cause of death from renal cancer. However, the molecular mechanism of tyrosine kinase binding protein (TYROBP) and sex-determining region Y Box-6 (SOX6) in the ccRCC was not precise.

**Methods:**

Bioinformatics analysis was performed to explore the hub role of TYROBP and SOX6 on the ccRCC. A total of 6 patients with clear cell renal cell carcinoma (ccRCC) were recruited. HE staining was performed to observe the pathology result of ccRCC. Immunohistochemistry and Immunofluorescence assay was made to detect the protein expression of TYROBP. Total RNA was extracted using TRIzol to examine the mRNA expression of TYROBP via the Real time quantitative polymerase chain reaction. The strong correlation between the expression of TYROBP and the survival time of ccRCC patients was performed by the BP neural network and support vector machine.

**Results:**

Compared with the control group, the expression of SOX6 was downregulated in the samples with ccRCC. However, the expression of TYROBP was higher in the samples with ccRCC than in the control group. Compared with the patients with high SOX6 expression, the patients with low SOX6 expression have a poor survival prognosis (HR=0.39, *P* < .05). However, the patients with high TYROBP expression have a shorter survival time than the patients with low TYROBP expression (HR=1.66, *P* < .05). The genes related with TYROBP and SOX6 are mainly enriched in the regulation of cell activation, leukocyte activation, negative regulation of cell activation, myeloid leukocyte activation, positive regulation of response to external stimulus, immune response-regulating signaling pathway. The interaction between TYROBP, SOX6, and kidney neoplasms was drawn, and the inference score of TYROBP and SOX6 on the kidney neoplasms was high.

**Conclusion:**

In conclusion, TYROBP is highly expressed in renal clear cell carcinoma, and when this molecule is highly expressed, the survival prognosis of renal carcinoma is poor. TYROBP and SOX6 may be potential targets for diagnosing and treating renal clear cell carcinoma.

## 1. Introduction

Renal cell carcinoma (RCC) is one of the most common urinary tumors globally, accounting for about 3% of all adult malignancies.^[1]^ The American Cancer Society estimates that there will be 73,750 new kidney and pelvic cancer cases and 14,830 deaths in the United States in 2020.^[2]^ RCC is a malignant tumor originating from renal tubular epithelium, and its incidence is increasing year by year.^[3]^ RCC has several subvariants, and approximately 70% of individuals are diagnosed with clear cell RCC (ccRCC).^[4]^ The early diagnosis of renal clear cell carcinoma is complex, and radical nephrectomy is usually required when it is found.

Meanwhile, conventional radiation and chemotherapy are ineffective against renal clear cell carcinoma, and the prognosis is frequently poor once cancer has spread. ccRCC is the most frequent subtype of renal cancer, accounting for around 75% of all histological forms of renal cancer. It is also the primary cause of renal cancer death.^[5]^ The prognosis of early localized clear cell carcinoma of the kidney is good after treatment, and the 5-year survival rate is more than 90%.^[6]^ Once distant metastasis occurs, survival time is drastically reduced, with an average survival period of 12–28 months after bone metastasis.^[7]^ Since 30%–50% of patients with clear cell carcinoma of the kidney lack clinical symptoms at an early stage, and about 20%–30% of patients are found to have distant metastasis at initial diagnosis, accurate early diagnosis is crucial for patients with clear cell carcinoma of the kidney.^[8]^ The etiology of renal clear cell carcinoma is unclear. The disease may be related to genetic factors, chromosome abnormalities, gene fusion, and other factors. Therefore, it is imperative to study the molecular mechanism of renal clear cell carcinoma.

TYROBP is a TYRO protein tyrosine kinase binding protein that regulates signal receptor binding, protein binding, microglia activation involved in immune response, positive regulation of gene expression, intracellular signal transduction, positive regulation of tumor necrosis factor biosynthesis, and apoptotic signaling pathways.^[9]^ TYROBP, formerly known as DAP12 and KARAP, encodes a transmembrane signal transduction peptide (tyrosine kinase binding protein). The gene encodes a transmembrane signaling polypeptide containing an activation motif based on the immune receptor tyrosine in its cytoplasmic domain.^[10]^ The proteins encoded could be members of the killer cell inhibitory receptor (KIR) family of membrane glycoproteins that activate signal transduction. This protein functions in signal transduction, bone modeling, brain myelination, and inflammation by binding to zeta chain-associated protein kinase 70 kDa (ZAP-70) and spleen tyrosine kinase.^[11]^ TYROBP is a regulatory protein of various activated receptors in natural killer cells, which activates small colloidal cells to recognize and phagocytose glioma cells.^[12]^ TYROBP can mediate natural killer (NK) cell activation with NK cell receptors such as KIR2DS2 and KLRD1/KLRC2 heterodimer and promote NK cell receptors KIR2DS1 and KIR2DS2 and KIR2DS4, and ensure its stability on the cell surface, enhance and maintain the cytotoxic effect of NK cells. TYROBP is a regulatory protein of various active receptors in NK cells, which can bind to active receptors in the form of noncovalent bonds to activate signal transduction and activate NK cells to perform effector functions.

Sex-determining region Y Box-6 (Sox6) is a hospital in the SOX family of transcription factors and plays an important role in early embryonic development, cell differentiation, and cell proliferation. In recent years, some scholars have found that SOX6 plays an important role in the occurrence and development of squamous cell carcinoma, liver cancer, chronic myeloid leukemia, and other tumors. Through the in-depth study of ovarian cancer, Li et al found that high expression of SOX6 can significantly inhibit the proliferation and invasion of ovarian cancer cell lines.

Furthermore, TYROBP could promote inflammation.^[13]^ The research found that TYROBP could promote the synthetic release of inflammatory factors, such as TNF-α, IL-10, IL-6, and MCP-1 in inflammatory diseases and magnify the inflammatory effect. The inflammatory response and mortality in the TYROBP gene knockout mice were significantly reduced.^[9]^ A study showed that serum levels of TNF-α and IL-6 in patients were significantly higher than those in healthy controls, and pathology also suggested MCP-1 infiltration in tissues, suggesting that TYROBP with high expression may be involved in the synthesis and release of such inflammatory factors. Elevated TYROBP expression predicts poor prognosis and high tumor immune infiltration in patients with low-grade glioma. Multivariate analysis showed elevated TYROBP expression is an independent risk factor for low-grade glioma.^[14]^ Therefore, we speculated that TYROBP might promote the proliferation of tumor cells by promoting inflammatory response. However, the specific molecular mechanism of TYROBP and SOX6 action in clear cell renal carcinoma remains unclear.

Therefore, in this study, we evaluated the expression pattern of TYROBP and SOX6 in renal transparent cell carcinoma tissues. We also investigated the clinical significance of TYROBP and SOX6 expression in patients with renal clear cell carcinoma.

## 2. Methods

### 2.1. Bioinformatics analysis for the TYROBP and SOX6 in the ccRCC

Based on The Cancer Genome Atlas (TCGA) database, the differently expressed genes (DEGs) related to the ccRCC were explored. The expression of TYROBP and SOX6 was compared between the tumor and control groups. Furthermore, the role of TYROBP and SOX6 on the tumor stage and survival was analyzed based on the TCGA. The relationship between TYROBP and SOX6 was explored. And the interaction of TYROBP and SOX6 and the ccRCC was described through the Comparative Toxicogenomics Database (CTD) database. The STRING was used to analyze the protein-protein interaction (PPI) network, and the visualization was made by Cytoscape. In addition, the hub genes were identified from the PPI network via the MCODE, degree, and MNC. Finally, the GO and KEGG analysis were made by the DAVID, Metascape.

### 2.2. Patients and ethics

A total of 6 patients with clear cell renal cell carcinoma (ccRCC) were recruited at the fourth hospital of Hebei medical university from March 2015 to June 2020. The patients are consecutive. And no patients were excluded according to the inclusion criteria and exclusion criteria.

Inclusion criteria: diagnosed as ccRCC; normal cardiopulmonary function; normal clotting function.

Exclusion criteria: poor pulmonary, cardiac, and liver function; Patients and their families did not agree to participate in the trial.

The Ethics Committee approved this study of the fourth hospital of Hebei medical university. Written informed consent was obtained from all patients.

### 2.3. Hematoxylin and eosin staining

ccRCC tissues of patients were obtained via surgery and preserved at −80°C immediately. First, the renal carcinoma and neighboring tissues were paraffin-embedded and sectioned, and the sections were washed in xylene for 10 min, anhydrous ethanol -95% alcohol -90% alcohol -80% alcohol -70% alcohol -distilled water. Second, the nucleus is stained with hematoxylin. The slices were stained with hematoxylin, rinsed with tap water, differentiated for several seconds with % alcohol hydrochloride, rinsed with tap water, returned to blue with 0.6% ammonia, and rinsed with running water. Finally, eosin is used to stain the cytoplasm. For staining, dip slices into the eosin solution for 1–3 minutes. Seal plate for dewatering the fourth. The slices were then dehydrated and transparent in 95% alcohol I 5min-95% alcohol II 5min-anhydrous ethanol I 5min-anhydrous ethanol II 5min-xylene I 5min-xylene II 5min. The slices were taken out from xylene, dried slightly, and sealed with neutral gum. A pathological image under a microscope (Axio Zoom V16, ZEISS, Germany) using Image-Pro Plus 5.0 software.

### 2.4. Immunohistochemistry

The slices of ccRCC tissues were placed in xylene, 95% ethanol, and 80% ethanol. Slices were rinsed with tap water 3 times and soaked in PBS for 5 min, 2 times total. PBS solution was soaked for 5 min, twice in total. Methanol hydrogen peroxide was soaked for 20 min. The slide was wiped and placed in a wet box, and the appropriate concentration of TYROBP monoclonal anti-antibody (dilution rate=1:2000, ab283679, Abcam) was added to the tissue drip so that the antibody entirely covered the tissue. The slide was placed in the refrigerator at 4°C overnight. The 50 µL immunochromogenic agent was added to the tissue to cover the tissue fully and incubated at room temperature for 25 min. The neutral resin seal sheet and read the sheet under a microscope.

### 2.5. Immunofluorescence assay for TYROBP

Washing 3 times with PBS (pH7.4) (5 min/time), immersed the renal sections in EDTA antigen retrieval buffer (pH 8.0) (Servicebio G1206, Wuhan, China) to make antigen retrieval. Treat with PBS (PH7.4) (3 times, 5 min/time), adding 3% BSA (Servicebio, G5001, Wuhan, China) to block nonspecific binding for 30 min. Throwing away the blocking solution, sections are incubated by TYROBP antibody (dilution rate=1:50, ab283679, Abcam) (overnight, at 4°C). Rewashing the sections with PBS (PH=7.4), fluorescent secondary antibodies responding to the primary antibodies were added (room temperature, 50 min, dark condition). Then incubated with DAPI solution (Servicebio, G1012, Wuhan, China) (room temperature, 10 min, darkness) to counterstaining the nucleus.

Finally, a spontaneous fluorescence quenching reagent (5 min) (Servicebio, G1221, Wuhan, China) was used to make spontaneous fluorescence quenching and seal the sections with an anti-fade mounting medium. The nuclei were blue (excitation wavelength 330–380 nm, emission 420 nm), and the positive expression was red or green, according to fluorescence microscopy (Nikon NIKON ECLIPSE C1) (FITC glows green by excitation wavelength 465–495 nm and emission 515–555nm; CY3 glows red by excitation wavelength 510–560 nm and emission 590 nm).

### 2.6. Real time quantitative polymerase chain reaction

Total RNA was extracted using the TRIzol^®^ (Beijing Biolab Technology Co., Ltd.) and reverse transcribed into cDNA with the Servicebio^®^RT First Strand cDNA Synthesis kit (cat. no. G3330, Wuhan Servicebio Biotechnology Co., Ltd.) for 60 min at 42°C. Terminate the reaction by heating at 70°C for 5 min. Real time quantitative polymerase chain reaction was performed in a Light Cycler^®^ 4800 System (Roche Diagnostics) with a specific set of primers to amplify secreted hub genes. Primers used were as follows: TYROBP-hF was CTGGGCGACAGAGTGAGA, and TYROBP-hR was GCCTGGGTGACAGAATGA. The thermocycling conditions used were as follows: 95°C for 15 s followed by 60°C for 60 s (a total of 30 cycles). The relative quantification units for each sample (relative quantification=2−Ct, where Ct stands for quantification cycle values) were calculated and displayed as fold changes in gene expression compared to the control group. As an endogenous control, GAPDH was employed.

### 2.7. Statistical methods

The data statistics are presented as sample size and percentage of the total. The Spearman-rho test was executed for the correlation analysis of SOX6 and TYROBP. Finally, we used the Kaplan–Meier method to explore overall survival (OS). All statistical analyses were conducted using SPSS software, version 24.0 (IBM Corp., Armonk, NY), and Matlab (MathWorks Inc., R2017a). A *P* value <.05 was considered statistically significant.

## 3. Results

### 3.1. Negative correlation between TYROBP and SOX6, and their role in the survival of ccRCC

**Figure 1.**
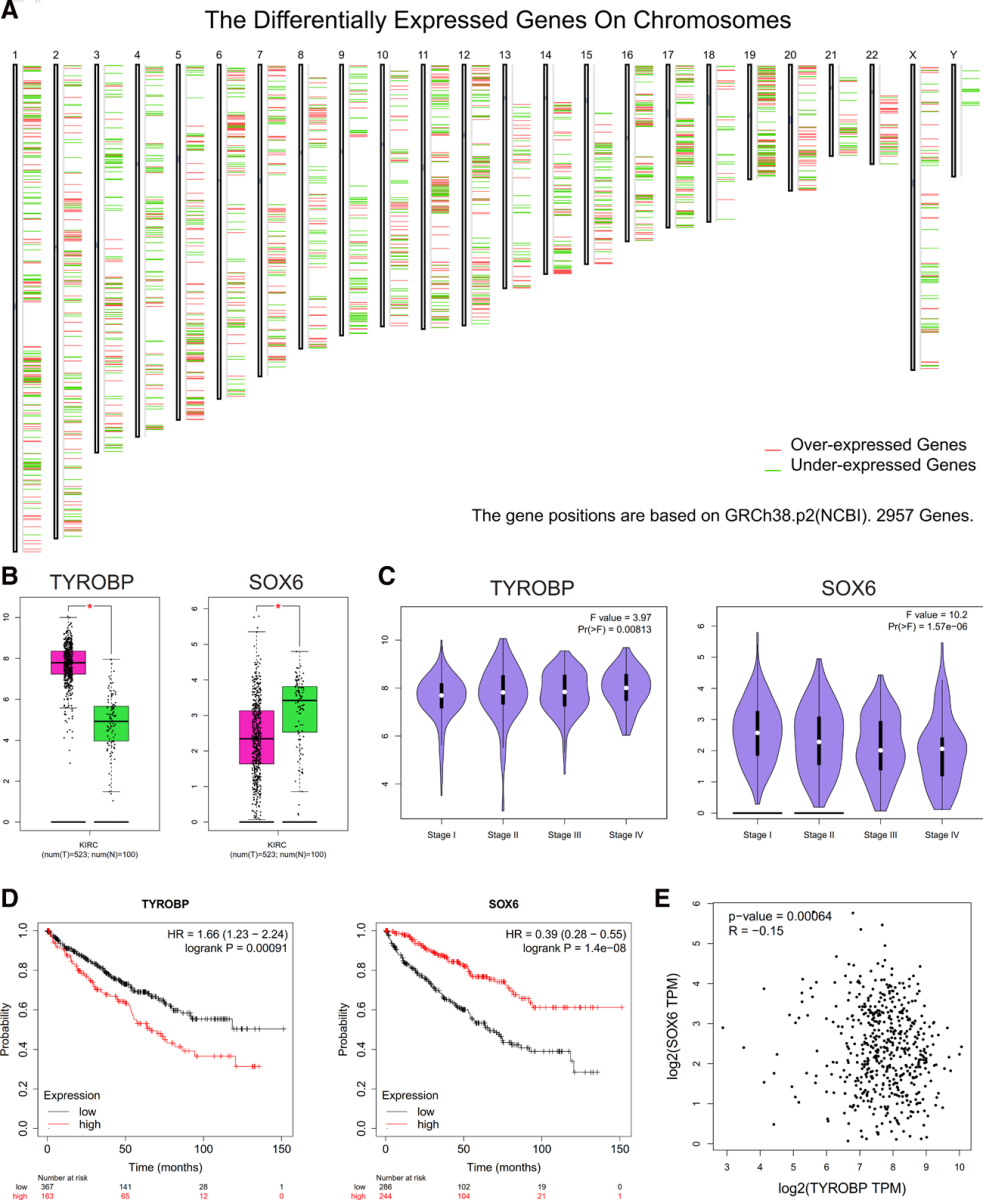
Negative correlation between TYROBP and SOX6, and their role in the survival of ccRCC. (A) The distribution of over and underexpressed genes on chromosomes presented the DEGs related to the ccRCC. (B) Expression of SOX6 and TYROBP. (C) Relationship between TYROBP, SOX6, and tumor stage. (D) Compared with the patients with high SOX6 expression, the patients with low SOX6 expression have a poor survival prognosis (HR=0.39, *P* < .05). However, the patients with high TYROBP expression have a shorter survival time than the patients with low TYROBP expression (HR=1.66, *P* < .05). (E) The expression of TYROBP was negatively related to SOX6. ccRCC = clear cell renal carcinoma, HR = hazard ratio, TYROBP = tyrosine kinase binding protein, SOX6, sex-determining region Y Box-6.

The distribution of over and underexpressed genes on chromosomes presented the DEGs related to the ccRCC (Fig. 1A). Compared with the control group, the expression of SOX6 was downregulated in the samples with ccRCC. However, the expression of TYROBP was higher in the samples with ccRCC than in the control group (Fig. 1B). There existed a negative relationship between the expression of SOX6 and tumor stage (*P* < .05). The higher expression of TYROBP was, the more severe the tumor stage was (*P* < .05, Fig. 1C). Compared with the patients with high SOX6 expression, the patients with low SOX6 expression have the poor survival prognosis (HR=0.39, *P* < .05). However, the patients with high TYROBP expression have shorter survival time than the patients with low TYROBP expression (HR=1.66, *P* < .05, Fig. 1D). The expression of TYROBP was negatively related to the SOX6 (*P* < .05, Fig. 1E).

### 3.2. Enrichment analysis via Metascape

**Figure 2.**
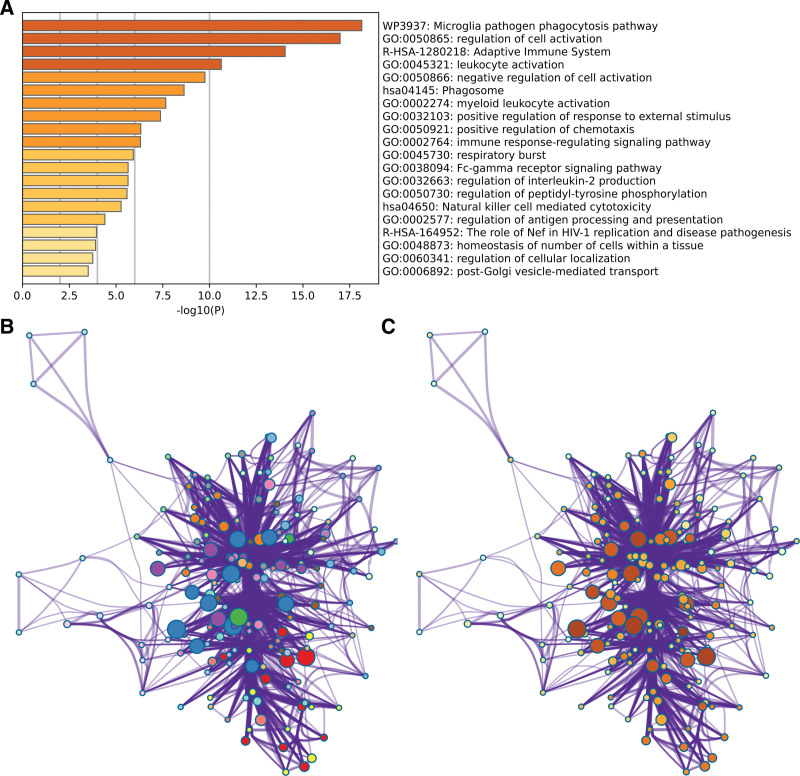
Enrichment analysis via Metascape.

Through the Metascape analysis, the genes related to TYROBP and SOX6 mainly enriched in the microglia pathogen phagocytosis pathway, regulation of cell activation, leukocyte activation, negative regulation of cell activation, myeloid leukocyte activation, positive regulation of response to external stimulus, immune response-regulating signaling pathway, Fc-gamma receptor signaling pathway, regulation of interleukin-2 production, regulation of antigen processing and presentation. And the clustering situation was also presented (*P* < .05). (Fig. 2)

### 3.3. GO and KEGG enrichment

**Figure 3.**
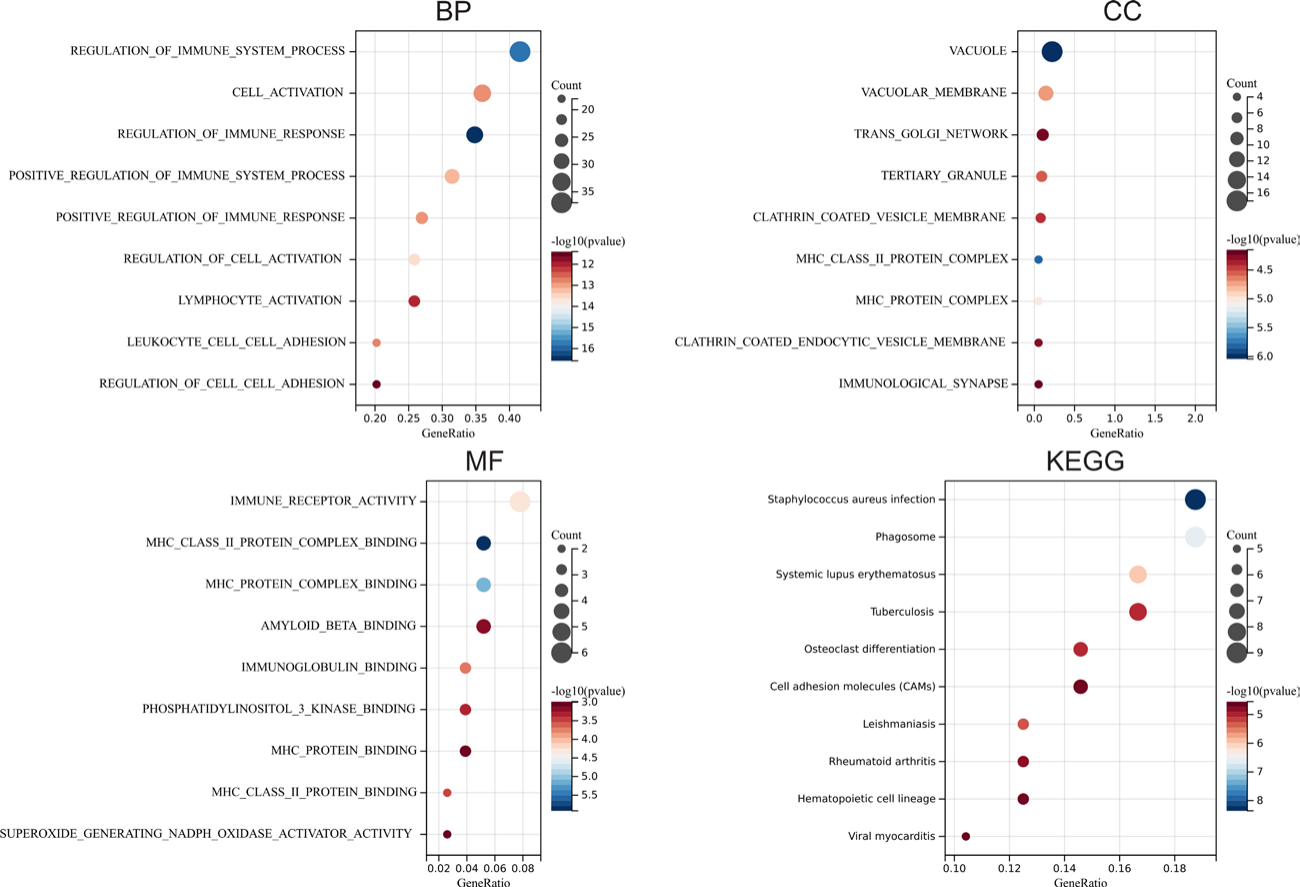
GO and KEGG enrichment.

Through the DAVID analysis, in the aspect of biological process (BP), the genes related to TYROBP and SOX6 are mainly enriched in the regulation of the immune system process, cell activation, regulation of immune response, and regulation of cell activation. In the aspect of cell component (CC), the genes related to TYROBP and SOX6 are mainly enriched in the vacuole, vacuolar membrane, trans-Golgi network, tertiary granule, clathrin-coated vesicle membrane, MHC protein complex, immunology synapse. In the aspect of molecular function (MF), the genes related to TYROBP and SOX6 are mainly enriched in immune receptor activity and immunoglobulin binding. In the aspect of KEGG, the genes related to TYROBP and SOX6 are mainly enriched in the staphylococcus aureus infection, phagosome, and cell adhesion molecules (Fig. 3).

### 3.4. Hub role of TYROBP in the ccRCC

**Figure 4.**
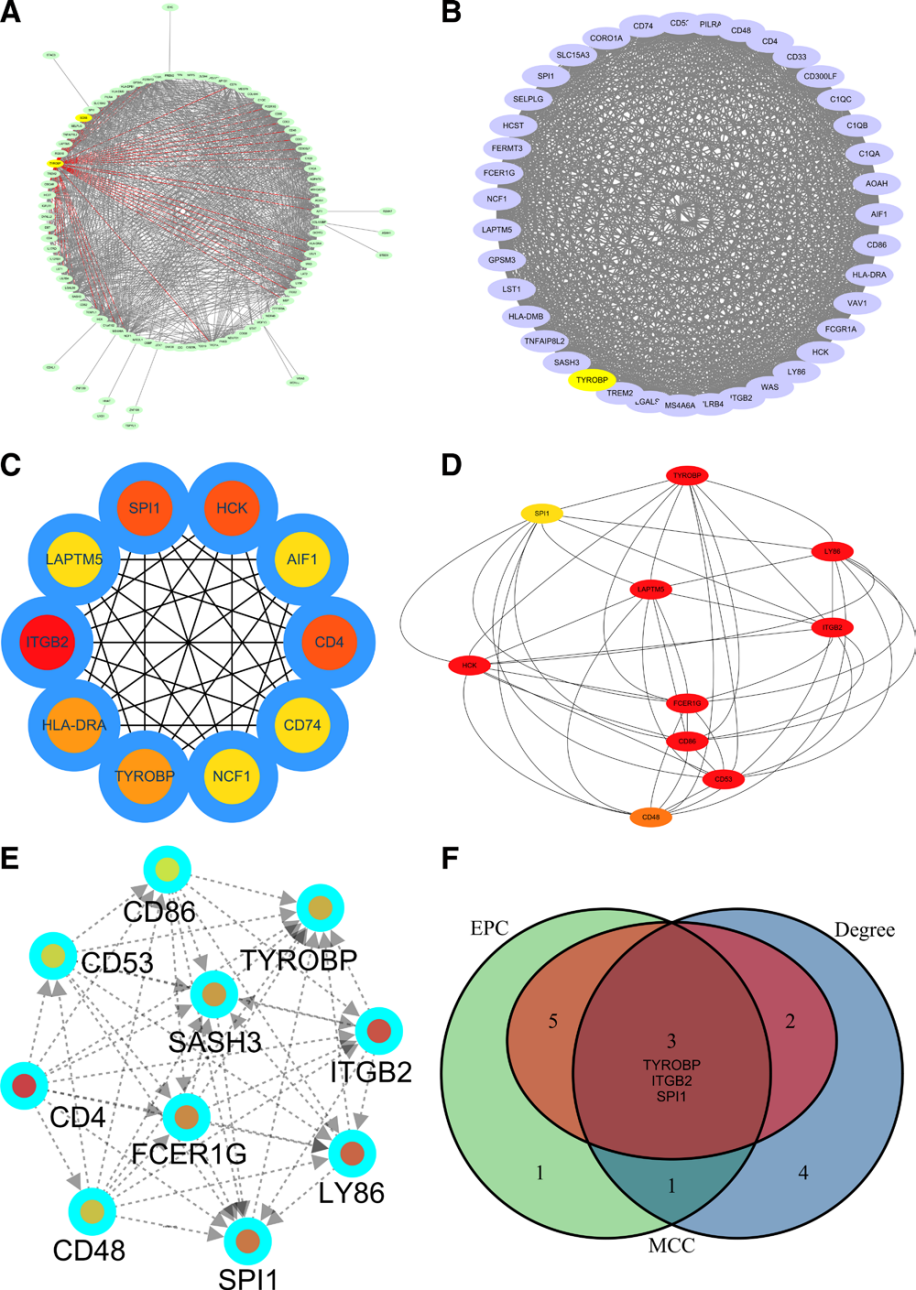
Hub role of TYROBP in the ccRCC. (A) PPI. (B) MCODE. (C) Degree. (D) MCC. (E) EPC. (F) Venn diagram was used to find the common hub genes among the Degree, MCC and EPC, and the TYROBP was identified as the most significant hub molecular. ccRCC = clear cell renal carcinoma, TYROBP = tyrosine kinase binding protein.

The PPI network presented the closed interaction among the genes related to the TYROBP and SOX6 (Fig. 4A). The MCODE was used to explore the hub network, and the TYROBP was the hub molecular in the module (Fig. 4B). Through the Degree method, the ten hub genes were identified (Fig. 4C), and the other ten hub genes were also identified by the MCC (Fig. 4D). In addition, another ten hub genes were found by the EPC (Fig. 4E). Finally, the Venn diagram was used to find the common hub genes among the Degree, MCC, and EPC, and the TYROBP was identified as the most significant hub molecular (Fig. 4F).

### 3.5. Analysis of immune infiltration for SOX6 and TYROBP on the ccRCC

**Figure 5.**
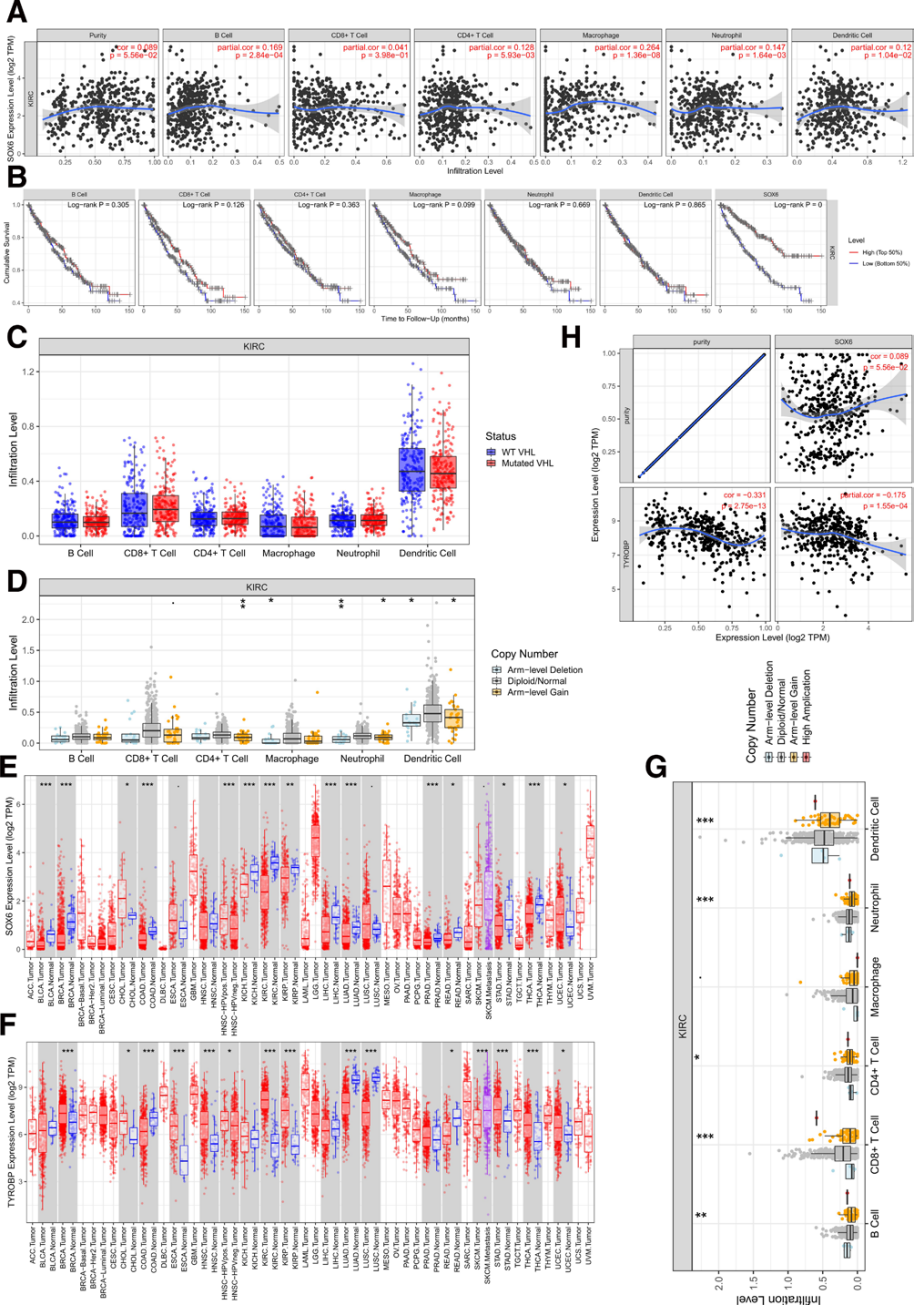
Analysis of immune infiltration for SOX6 and TYROBP on the ccRCC. ccRCC = clear cell renal carcinoma, TYROBP = tyrosine kinase binding protein, SOX6, sex-determining region Y Box-6.

The relationship between SOX6 expression and proportion of immune infiltration was presented in Fig. 5A. The relationship between clinical outcome, immune cell infiltration, and SOX6 gene expression is shown in Fig. 5B, and the SOX6 expression was significantly related to the dendritic cell and the survival of the ccRCC patients (Fig. 5B). This module shows the correlation between SOX6 gene mutation and immune infiltration and tests whether SOX6 gene mutation affects the infiltration ratio of immune cells (Fig. 5C). This module explores the relationship between somatic copy number variation of SOX6 and immune infiltration. We applied GISTIC2.0 data to examine the effect of different gene copy statuses on immune infiltration compared with normal tissues (Fig. 5D). The differential expression of the SOX6 gene in tumors of multiple cancer species and normal tissues is shown in Fig. 5E. The differential expression of the TYROBP gene in tumors of multiple cancer species and normal tissues is shown in Fig. 5F. This module explores the relationship between somatic copy number variation of TYROBP and immune infiltration. We applied GISTIC2.0 data to examine the effect of different gene copy statuses on immune infiltration compared with normal tissues (Fig. 5G). The SOX6 was negatively related to the TYROBP in the ccRCC disease (*P* < .05, Fig. 5H).

### 3.6. CTD analysis

**Figure 6.**
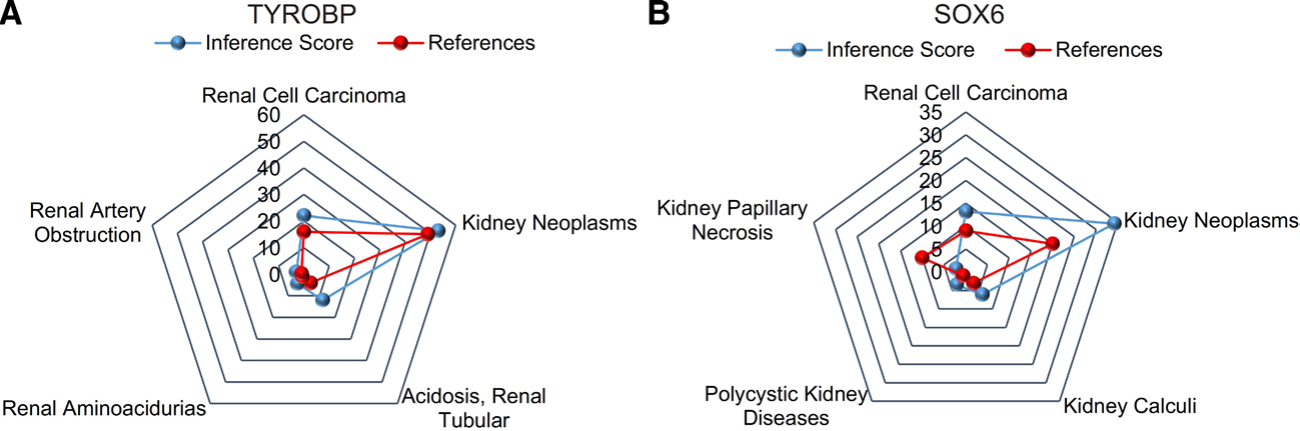
The interaction between TYROBP, SOX6, and kidney neoplasms was drawn, and the inference score of TYROBP and SOX6 on the kidney neoplasms was high. (A) TYROBP. (B) SOX6. TYROBP = tyrosine kinase binding protein, SOX6, sex-determining region Y Box-6.

The interaction between TYROBP, SOX6, and kidney neoplasms was drawn, and the inference score of TYROBP and SOX6 on the kidney neoplasms was high (Fig. 6).

### 3.7. Pathological morphologic changes of ccRCC via the HE staining

**Figure 7.**
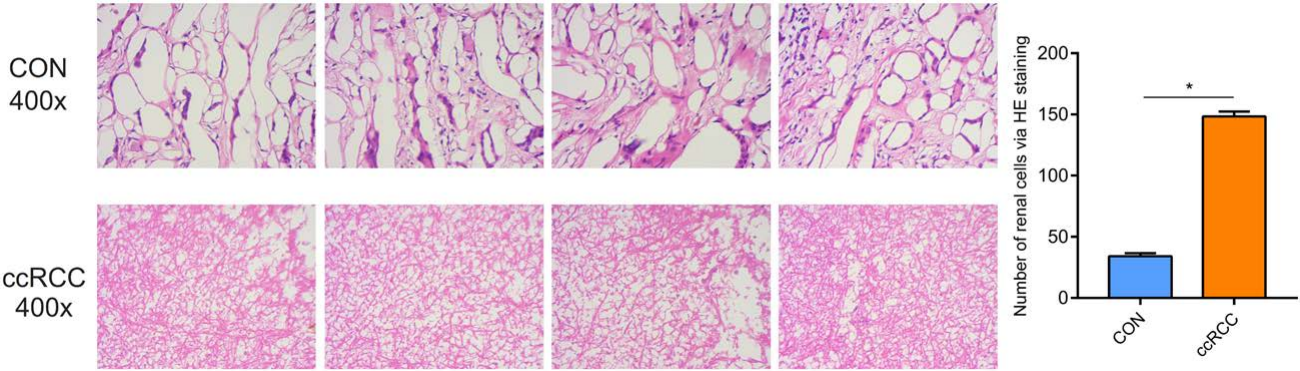
The protein expression of TYROBP in the ccRCC and control tissues via immunohistochemical assay. ccRCC = clear cell renal carcinoma, TYROBP = tyrosine kinase binding protein.

The number of renal cells via HE staining in the control tissues was lower than the ccRCC (*P* < 0.05). And in the ccRCC, there exist changes in cell morphology and structure, and immature cells are more common (Fig. 7).

### 3.8. The protein expression of TYROBP in the ccRCC

**Figure 8.**
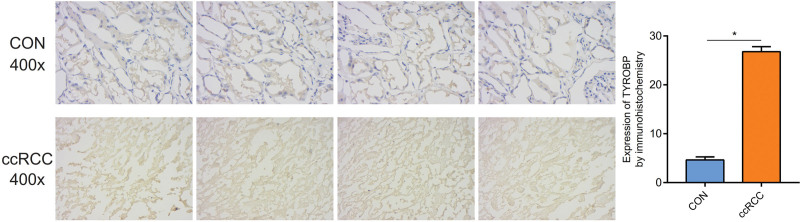
Verification of protein expression of TYROBP by immunofluorescence. TYROBP = tyrosine kinase binding protein.

**Figure 9.**
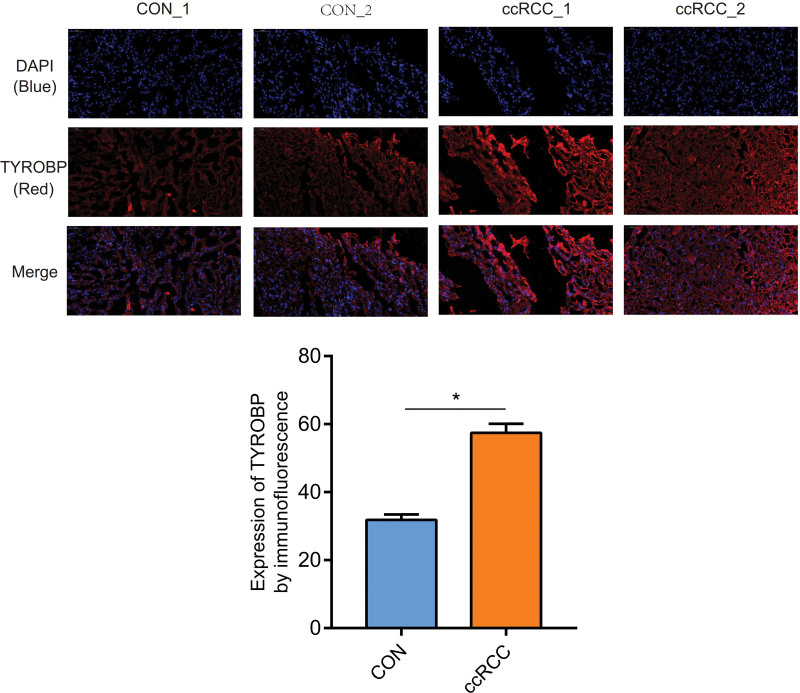
Strong correlation between the expression of TYROBP and the survival time of ccRCC patients based on the BP neural network and support vector machine (SVM). ccRCC = clear cell renal carcinoma, TYROBP = tyrosine kinase binding protein.

The immunohistochemical assay showed that the protein expression of TYROBP in the ccRCC was higher than in the control tissues (*P* < .05). In the ccRCC, the area of yellow presented the expression of TYROBP, and there were obvious yellow plots in the ccRCC tissue (Fig. 8). Furthermore, verification of protein expression of TYROBP by the immunofluorescence was also performed. In the immunofluorescence assay, the area of red presented the expression of TYROBP, which manifested that protein expression of TYROBP in the control tissues was significantly lower than in the ccRCC tissues (Fig. 9).

## 4. Discussion

Renal parenchymal carcinoma is adenocarcinoma derived from renal tubular epithelial cells. Among all renal cell carcinoma tissue subtypes, renal clear cell carcinoma accounts for more than 80%. In addition, renal clear cell carcinoma is the tissue subtype with the highest mortality, invasion rate, and metastasis rate and has the most vigorous resistance to traditional chemotherapy and radiotherapy.^[5,15]^ Renal clear cell carcinoma accounts for about 3% of the total number of human malignant tumors, and the incidence of renal clear cell carcinoma has been increasing in recent years. Nearly 50% of patients are found to be advanced.^[16,17]^ Clear cell carcinoma of the kidney is usually asymptomatic in its early stages or only presents with systemic symptoms such as fever and fatigue, and tumor size increases. Renal clear cell carcinoma has the highest mortality among all urinary system tumors, and lymphatic system metastasis is one of the main reasons for poor clinical treatment. Therefore, it is essential for the early detection and judgment of renal clear cell carcinoma.^[18]^ It is crucial to explore the molecular mechanism of renal clear cell carcinoma to research targeted drugs. The main results of this study were that TYROBP was highly expressed in renal clear cell carcinoma. When the molecule was highly expressed, the survival prognosis of renal carcinoma was poor.

TYROBP, also known as DAP12, is a microglial transmembrane marker polypeptide. Its cytoplasmic domain contains an activation motif (ITAM) based on immune receptor tyrosine, which is a ligand of immune receptors including myeloid triggering receptor 2, complement receptor 3, and signal regulatory protein TYROBP binds to corresponding receptors to phosphorylation of ITAM, and then plays its role through a series of signal transduction pathways. TYROBP can bind to SIRPB-1 and TREM-1 to mediate the activation of myeloid cells such as neutrophils, monocytes, and dendritic cells and the upregulation of chemokine receptors CCR7 to promote neutrophils degranulation.^[13]^

TYROBP is a TYRO protein tyrosine kinase binding protein that regulates signal receptor binding, protein binding, microglia activation involved in immune response, positive regulation of gene expression, intracellular signal transduction, positive regulation of tumor necrosis factor biosynthesis, and apoptotic signaling pathways. The abnormal expression of the TYROBP gene is also involved in the occurrence and development of many diseases, among which TYROBP is involved in the pathogenesis of inflammatory disease.^[14]^

TYROBP is also involved in the development and progression of tumors. Kalaigi suggested that the mechanisms involved in the metastasis and spread of circulating tumor cells (CTCs) remain clarified due to different tumors’ biological characteristics and heterogeneity. However, the authors found that 75% of breast cancer patients with CTCs were TYROBP positive. These results suggest that TYROBP can be used as a novel biomarker and a potential therapeutic target for eliminating CTC.^[9,10,12,14,19,20]^

Through data analysis of RCC in Gene Expression Omnibus database and application of related feature subsets method in DEGs essential gene selection, several critical genes related to the pathogenesis of renal cell carcinoma were found. In addition, the high expression of TYROBP in patients with renal cell carcinoma is associated with poor prognosis, suggesting that TYROBP may be involved in the occurrence and development of renal cell carcinoma and can be used as a target for diagnosis and treatment. We found that compared with normal kidney tissue, the expression of TYROBP in tumor tissues of ccRCC patients was significantly upregulated. In contrast, the survival prognosis of ccRCC patients with high expression of TYROBP was poor. We speculated that TYROBP might be involved in the occurrence and development of ccRCC by affecting immune function, inflammation, and apoptosis.^[9]^

The PPI network was constructed with 660 differential genes screened out, and the score of network nodes was calculated. The 15 most critical genes in ccRCC were screened out from high to low. The formation and analysis showed that the expression levels of CCL5, TYROBP, LILRB2, and MMP9 were closely related to the survival rate of patients. It was suggested that CCL5, TYROBP, LILRB2, and MMP9 may play an essential role in the pathogenesis and development of ccRCC and may have potential clinical diagnostic and therapeutic value.^[9]^ The survival rate of patients with low expression of TYROBP and LILRB2 genes was significantly higher than those with increased expression of LILRB2 and TYROBP genes. The expression of TYROBP and LILRB2 may have important significance for the diagnosis, treatment, and prognosis of ccRCC.^[19]^ There are differentially expressed genes between cancer and normal kidney tissue in renal clear cell carcinoma patients. Through further analysis, we found that TYROBP, FCGR2B, TYROBP, LY86, and TLR2 genes were highly expressed in ccRCC carcinoma tissues.^[21,22]^

High expression of TYROBP was associated with the low survival rate of ccRCC, was closely related to immune cell infiltration, and was co-expressed with PD-1 and CTLA-4. In conclusion, TYROBP may have diagnostic and therapeutic potential for ccRCC.^[23]^ The relationship between TYROBP and immune cell infiltration showed that the high expression of TYROBP was related to the high infiltration rate of immune cells, including B cells, CD8+T cells, CD4+T macrophages, neutrophils, and dendritic cells. The differentially expressed genes (DEGs) are mainly involved in glycolysis/gluconeogenesis, PPAR signaling pathway, complement, and coagulation cascade reactions.^[24]^ This suggests that ccRCC is closely related to metabolism and immune regulation.^[25]^ Many studies have shown that carcinogenesis may be closely associated with metabolism, confirming our research’s general direction. Increased glycolysis is a marker of malignancy and is associated with aggressiveness and poor prognosis. Tumor cells use aerobic glycolysis to meet energy and membrane structure requirements to achieve the Warburg effect, especially in ccRCCs.^[26]^

Survival analysis showed that 8 downregulated hub genes (HRG, FABP1, ALDOB, PCK1, HAO2, CASR, PLG, HMGCS2) and 2 upregulated hub genes (SERPINE1 and TYROBP) might be the essential genes of ccRCC. Previous studies identified TYROBP as a pivotal gene in the progression of ccRCC through WGCNA analysis, suggesting that TYROBP is closely related to clinical traits and important biological processes.^[27]^ TYROBP and its related genes are mainly involved in immunomodulatory mechanisms such as NK cell-mediated cytotoxicity. TYROBP may promote tumor progression by interacting with immune cells. It has recently been reported that cell fusion contributes to the spread of cancer, and TYROBP is critical for the fusion of macrophages. Tumor cells in the bone microenvironment stimulate the recruitment and activation of osteoclasts and osteoblasts. The tumor microenvironment promotes tumor proliferation and determines the perfusion site of metastasis. TYROBP was highly expressed in breast cancer cells and significantly associated with bone metastasis and poor prognosis.^[28]^ The TYROBP/ITAM pathway may be involved in breast cancer bone metastasis. ccRCC has been reported to have a high potential for bone metastasis in the form of osteoclasts. Whether the increased expression of TYROBP is related to bone metastasis of ccRCC is the direction of our further research. Therefore, it is speculated that TYROBP may play an essential role in developing renal clear cell carcinoma.^[29]^

The above literature review is consistent with our results: TYROBP is highly expressed in renal clear cell carcinoma, and when this molecule is highly expressed, the survival prognosis of renal carcinoma is poor. Despite the rigorous bioinformatics analysis in this paper, some shortcomings remain. In this study, no animal experiments of gene overexpression or knockout were conducted to verify its function further. Therefore, we should carry out an in-depth exploration of this aspect in future research.

In conclusion, TYROBP is highly expressed in renal clear cell carcinoma, and when this molecule is highly expressed, the survival prognosis of renal carcinoma is poor. TYROBP and SOX6 may be potential targets for diagnosing and treating renal clear cell carcinoma.

## Author contributions

Feng Li experimented and was a significant contributor in writing. Xian-qiang Lv was involved in critically revising the manuscript for important intellectual content. Xian-qiang Lv made substantial contributions to research conception. Feng Li designed the draft of the research process. Kai-bo Zhang and Xu Guo analyzed the data. Xian-qiang Lv was a significant contributor in submitting the manuscript. Feng Li gives a technical support. All authors read and approved the final manuscript.
